# Non-Dipping Blood Pressure in Pediatric Hypertension: A Narrative Review

**DOI:** 10.31083/RCM48601

**Published:** 2026-05-06

**Authors:** Elisa Gherbesi, Andrea Faggiano, Stefano Carugo, Guido Grassi, Marijana Tadic, Cesare Cuspidi

**Affiliations:** ^1^Department of Cardio-Thoracic-Vascular Diseases, Foundation IRCCS Ca’ Granda Ospedale Maggiore Policlinico, 20122 Milano, Italy; ^2^Department of Clinical Sciences and Community Health, University of Milano, 20122 Milano, Italy; ^3^Department of Medicine and Surgery, University of Milano-Bicocca, 20126 Milano, Italy; ^4^University Heart Center Ulm, University Ulm, 89081 Ulm, Germany

**Keywords:** ambulatory blood pressure monitoring, hypertension, child, left ventricular hypertrophy

## Abstract

Ambulatory blood pressure monitoring (ABPM) provides a unique opportunity to assess day–night blood pressure (BP) variability. Individuals whose night-time BP is 10–20% lower than the subsequent daytime BP values are defined as dippers, whereas those with a nocturnal BP decrease of <10% are considered non-dippers. A non-dipping (ND) pattern has been shown to occur in a substantial proportion of adults with hypertension and is influenced by age, sex, ethnicity, and comorbidities. More importantly, the ND pattern has been reported to adversely affect hypertension-mediated organ damage (HMOD) and cardiovascular prognosis, due to a greater nighttime pressure overload that promotes more severe cardiovascular damage. Notably, current evidence on alterations in circadian BP rhythm in children and adolescents with hypertension remains scarce. Therefore, this narrative review aimed to analyze the available literature on this important topic and to provide updated, comprehensive information on the prevalence of the ND pattern and its association with cardiac and extracardiac HMOD markers. The prevalence of ND patterns (based on data from 16 studies) ranged from 35% to 72% (mean 59%). Regarding the association between ND and HMOD, particularly echocardiographic left ventricular hypertrophy, results have been mixed, leaving uncertainty about whether a blunted nocturnal BP decline in pediatric hypertension contributes to HMOD. Thus, large prospective studies are needed to improve definitions of the clinical significance of alterations in BP circadian rhythm in children and adolescents with hypertension, focusing on methodological issues not yet fully addressed, such as the use of pediatric diagnostic criteria and the reproducibility of the ND pattern over time.

## 1. Introduction 

One of the most important requirements for the diagnosis and clinical management of individuals with blood pressure (BP) values above the normal threshold defined by hypertension guidelines is represented by an accurate measurement performed in a medical environment under controlled conditions in order to reduce as much as possible methodological pitfalls and overestimation of the real BP level related to an alarm reaction [1, 2, 3]. This is because a significant amount of evidence on the adverse prognostic significance of hypertension as well as the protective benefit of antihypertensive treatment in the adult and elderly populations has historically been obtained from prospective observational studies and randomized intervention trials based on office BP measurements [4, 5].

Although conventional office BP measurement represents the standard BP measurement method, the implementation of out-of-office BP measurements (24-hour ambulatory and home BP) to improve diagnostic accuracy is strongly recommended by contemporary guidelines for adult individuals, as it has been shown to offer additional important advantages. Greater BP reproducibility, identification of abnormal BP phenotypes such as white coat, masked and sustained hypertension, and a stronger prognostic value are the key features of mutual integration between office and out-of-office BP [6, 7, 8]. In particular, the 24-hour (h) ambulatory BP monitoring (ABPM) has the peculiar capability to record BP changes throughout the day and provide information on the extent of the physiological drop in BP at night, allowing individuals to be classified into dippers and non-dippers [9, 10, 11].

In the last four decades, in parallel with the notion of different prognostic significance of abnormal BP phenotypes resulting from the combined assessment of in- and out-of-office BP, a large body of information has emerged about the excess cardiovascular (CV) risk linked to a blunted nocturnal BP fall in the adult hypertensive setting. Indeed, numerous cross-sectional studies have highlighted a relationship between non-dipping (ND) pattern and cardiac and extracardiac hypertension-mediated organ damage (HMOD), and prospective longitudinal studies have revealed an independent association between this pattern and new-onset HMOD and an increased incidence of fatal and non-fatal CV events [12, 13].

Some of the benefits resulting from the implementation of ABPM in everyday clinical practice have been confirmed in children, and in addition to office BP measurement, this technique offers a more comprehensive, accurate, and reliable assessment of BP status, leading to more precise diagnosis and treatment decision-making in children and adolescents [14]. Therefore, consistent with recommendations for adult hypertension, available pediatric guidelines and expert consensus acknowledge the importance of 24-h ABPM, suggesting a much wider use for diagnostic and therapeutic purposes [15, 16]. However, many gaps remain in the use and interpretation of ABPM in children and adolescents. This was evidenced by the repeated updates released by the guidelines on this topic. For instance, the 2022 American Heart Association (AHA) pediatric ABPM guidelines removed BP classification based on BP loads, recommending that each patient should be categorized according to four BP phenotypes: normotension, white-coat hypertension, masked hypertension, and ambulatory hypertension [17]. Nonetheless, clinical interpretation of ABPM in the pediatric setting remains problematic owing to the limited number of reference values. In addition, it should not be ignored that children tolerate less well and have lower compliance during ABPM than adults; both of these variables make the interpretation of the entire 24-h recording and, particularly, the night-time period, difficult.

Starting from this background and given the paucity of data on circadian variations of BP in pediatric hypertension, we carried out a systematic review on this topic in order to provide comprehensive and updated information on the prevalence of the ND pattern and its association with HMOD in children and adolescents with hypertension.

## 2. Literature Search

This review was prepared in accordance with the Narrative Review Checklist (available at https://legacyfileshare.elsevier.com/promis_misc/ANDJ%20Narrative%20Review%20Checklist.pdf) and prospectively registered with the International Prospective Register of Systematic Reviews (PROSPERO, unique identifier: ID 1048879). The medical literature was reviewed to identify articles evaluating circadian variations in BP (i.e., non-dipping status) in pediatric hypertension. A computerized search was performed using PubMed, OVID, and Cochrane Library databases from inception to June 30th 2025. Studies were identified by using the following search terms: “dipping”, “non-dipping”, “ambulatory blood pressure monitoring”, “pediatric hypertension”, “circadian blood pressure rhythm”, “night blood pressure fall”.

Checks of the reference lists of the selected papers and pertinent reviews complemented the electronic search. Data were examined and extracted by two independent investigators (EG and CC). In case of no agreement on a specific record, the full text of the study was analyzed by all reviewers to establish eligibility according to the inclusion criteria mentioned below. The main inclusion criteria were: (I) articles published in peer-reviewed journals; (II) studies providing data on ND prevalence in pediatric hypertension; and (III) satisfactory quality paper according to the Newcastle-Ottawa Scale (i.e., score >6).

## 3. Main Findings

**Fig. 1.**
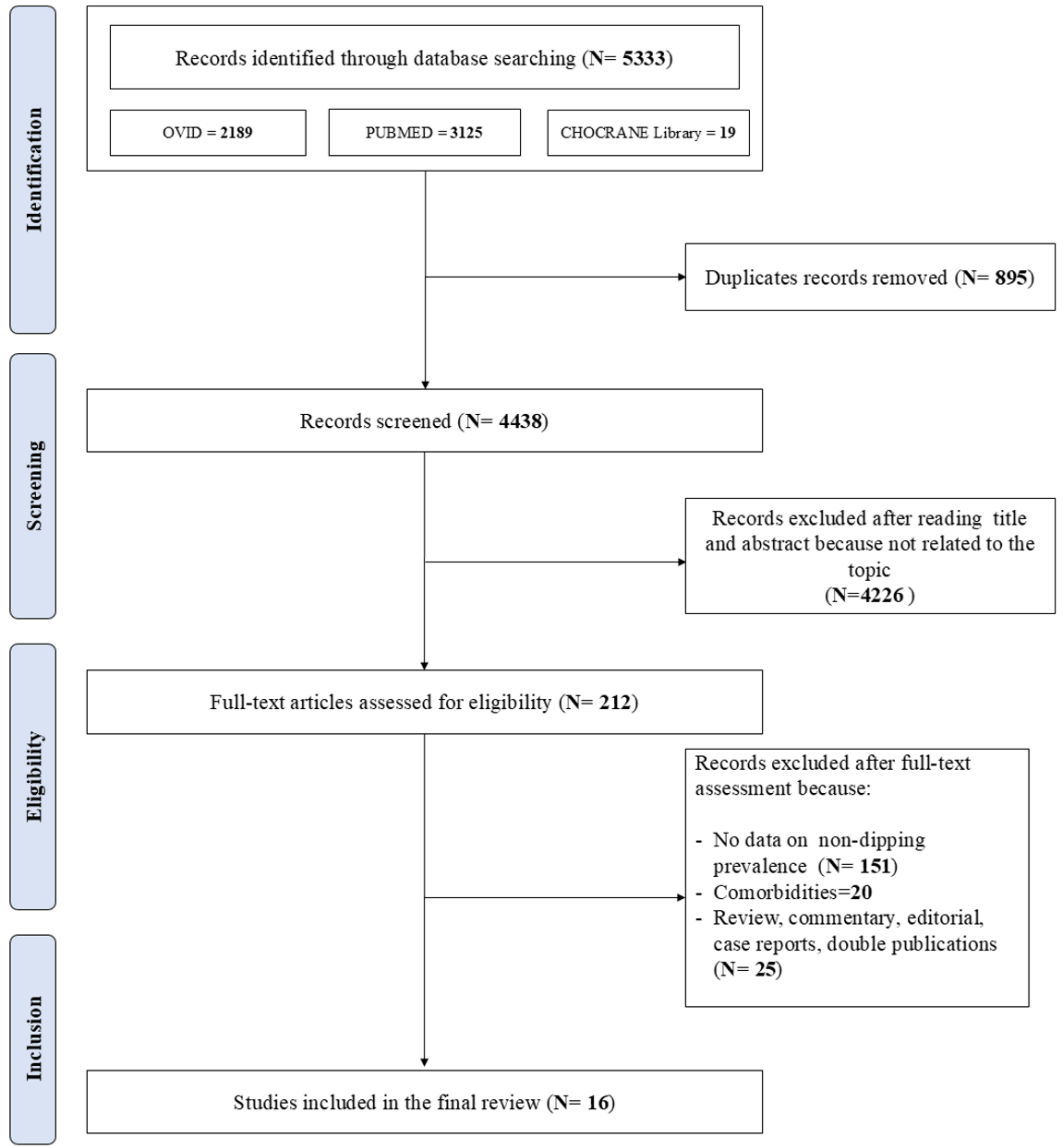
**Schematic flow-chart for the selection of studies**.

The first literature search identified 5333 papers. After duplicate records were removed (n = 895), the initial screening of titles and abstracts excluded 4226 out of 4438 studies as they were not related to the topic. Therefore, 212 studies were reviewed; of these, 151 did not report categorical data on the ND pattern,20 included patients with comorbidities , 25 were reviews, commentaries, editorials , case reports and double publications. Sixteen studies, including participants with primary or secondary hypertension, were included in the final review [18, 19, 20, 21, 22, 23, 24, 25, 26, 27, 28, 29, 30, 31, 32, 33] (Fig. 1). The Newcastle-Ottawa Score used to assess the quality of the studies ranged from 7 to 9, with a mean score of 7.3. No study was excluded because of its limited quality.

### 3.1 Characteristics of the Studies

Overall, 2204 children/adolescents with primary or secondary hypertension and normotensive controls were included in 16 studies (sample size ranging from 30 to 539 participants) performed in three continental areas (Asia = 6, Europe = 5, North America = 4). One of the selected studies reported the results collected in the context of international multicenter research [24].

Table 1 (Ref. [18, 19, 20, 21, 22, 23, 24, 25, 26, 27, 28, 29, 30, 31, 32, 33]) shows the demographic and clinical characteristics of the participants of the selected studies, such as sample size, setting, mean age, sex distribution, and research purpose of the single studies.

**Table 1.** **Summary of studies providing data of prevalence of non-dipping pattern in the setting of pediatric hypertension**.

Author (reference), year publication	Sample size HTN (n)	Type of study	Age (yrs)	Comorbidities	Aims of the studies	Main results
	Male (%)	Setting				
Seeman *et al* [18], 2005	145 (68%)	CS Retrospective Study	15.7 ± 3.4; 11.8 ± 4.2	Renal parenchymal and renovascular disease	Prevalence ND in secondary HTN	Secondary HTN was characterized by reduced nocturnal BP dip and increased nighttime BP load. A ND pattern, especially diastolic, showed very high specificity for secondary HTN.
		Primary and secondary HTN				
Krzych and Szydlowski [19], 2009	106 (73%)	CS Retrospective Study	14.9 ± 2.5	No	Determinants of ND	ND was associated with higher nocturnal BP and BP load, reflecting more severe HTN. Male sex showed a trend toward increased ND risk, with no other clinical determinants identified.
		Primary HTN				
Valent-Morić *et al* [20], 2012	139 (68%)	CS Retrospective Study	14.1 (4.1–19.0)	No	Ambulatory BP phenotypes	ABPM identified HTN in ∼77% of children. ND was frequent across primary and secondary HTN with no differences in BP load.
		Primary and secondary HTN				
Conkar *et al* [21], 2015	82 (57%)	CS Prospective Study	13.3 ± 4.1	No	ND and HMOD	In primary HTN, daytime SBP load correlated with vascular damage markers, whereas ND per se was not independently associated with target organ damage.
		Primary HTN				
Seeman *et al* [22], 2016	114 (60%)	CS Retrospective Study	15.3 (3.8–18.9)	Renal parenchymal disease (70%)	ND and LVH	ND was not associated with increased left ventricular hypertrophy prevalence. Left ventricular mas was independently related to nocturnal BP levels, rather than dipping status.
		Primary and secondary HTN		Obesity (21%)		
Cetin and Kavaz Tufan [23], 2019	89 (50%)	CS Retrospective Study	11.6 ± 3.7	CKD stage 1	ND and Inflammatory markers	ND was associated with increased platelet activation and systemic inflammation, suggesting an elevated atherosclerotic risk.
		HTN and CKD				
Shilly *et al* [24], 2019	119 (71%)	CS Retrospective Study	14.0 ± 3.3	No	ND and LVH	In primary HTN, higher awake and sleep SBP load predicted concentric hypertrophy, while ND was not associated with LV geometry.
		Primary HTN				
Düzova *et al* [25], 2019	162 (52%)	CS Prospective Multicenter Study	12.5 ± 5.2	CKD stage III–IV	ND and HMOD	In CKD, cardiovascular damage was driven by nocturnal HTN rather than ND status.
		HTN and CKD				
Cilsal [26], 2020	30 (60%)	CS Retrospective Case-control, Study	14.4 ± 3.0	No	ND, BP variability, and HMOD	In essential HTN, ND was not associated with higher left ventricular mas, but was characterized by reduced autonomic modulation and increased arterial stiffness.
		Primary HTN				
Bakhoum *et al* [27], 2021	77 (65%)	CS Retrospective Study	14.2 ± 3.5	Proteinuria or MA	ND, Proteinuria and MA	ND was independently associated with higher proteinuria, with increasing urine protein-to-creatinine ratio linked to reduced systolic and diastolic dipping.
		HTN with proteinuria or MA				
Wu *et al* [28], 2022	425 (78%)	CS Retrospective Case-control, Study	13.0 ± 2.3	Obesity (71%)	ND and LVH	ND was not associated with left ventricular hypertrophy in essential HTN, rather driven by higher nighttime BP and increased 24-h SBP load, independent of dipping status.
		Primary HTN				
Szyszka *et al* [29], 2022	112 (70%)	CS Prospective Study	14.7 ± 2.1	No	Factors associated to ND	ND was common in primary HTN and was associated with higher left ventricular mass, while extreme dipping was linked to increased arterial stiffness.
		Primary HTN				
Pagi *et al* [30], 2023	501 (61%)	CS Prospective Multicenter Study	12.7 ± 3.8	CKD stage 1–5	Racial diversity in ABPM	In CKD, ND was more prevalent in Black patients, with ∼2-fold higher odds of systolic and diastolic ND compared with White peers, alongside higher nocturnal BP and BP load.
		HTN and CKD				
Kogon *et al* [31], 2024	539 (61%)	CS Retrospective Study	14.6 ± 3.0	Obesity (32%)	ND and sleep	Longer sleep duration and excessive sleep were associated with reduced nocturnal dipping and higher odds of blunted ND, despite lower daytime BP parameters.
		NTN and HTN				
Sun *et al* [32], 2025	125 (79%)	CS Retrospective Study	12.9 ± 0.20	Obesity (% n.a.)	Factors associated to ND	A ND pattern was frequent in essential HTN and was associated with a higher prevalence of left ventricular hypertrophy. Higher platelet activation, inflammation, and triglyceride levels independently predicted ND status.
		Primary HTN				
Taner *et al* [33], 2025	140 (64%)	CS Retrospective Study	13.9 ± 2.6	Obesity (61.4%)	ABPM obesity and hyperuricemia	A ND pattern was very common in primary HTN and was associated with a higher prevalence of left ventricular hypertrophy, independent of obesity and uric acid levels.
		HTN and CKD				

HTN, hypertension; ND, non-dipping; HMOD, hypertension-mediated organ damage; CKD, chronic kidney disease; MA, microalbuminuria; ABPM, ambulatory blood pressure monitoring; CS, cross-sectional; SBP, systolic blood pressure; BP, blood pressure; LVH, left ventricular hypertrophy; n.a., not available. Data are presented as absolute numbers, percentages, mean ± SD.

The mean age range was 11.6–15.7 years. Male sex was prevalent in all studies, except for one, which ranged from 52 to 78%. The selected studies included hypertensive children/adolescents with different clinical conditions such as primary hypertension, hypertension associated with chronic kidney disease, and secondary hypertension. The research objective of the studies, providing information on the dipping/ND status as a categorical variable, focused on the association of the ND pattern with different HMOD markers and/or its potential correlates/determinants, such as obesity, ethnicity, inflammatory factors, and sleep. Notably, all studies were characterized by a predominantly retrospective cross-sectional design, which a priori limits the presence of a robust causal link between altered circadian blood pressure rhythm and organ damage.

### 3.2 Prevalence of ND Pattern

**Fig. 2.**
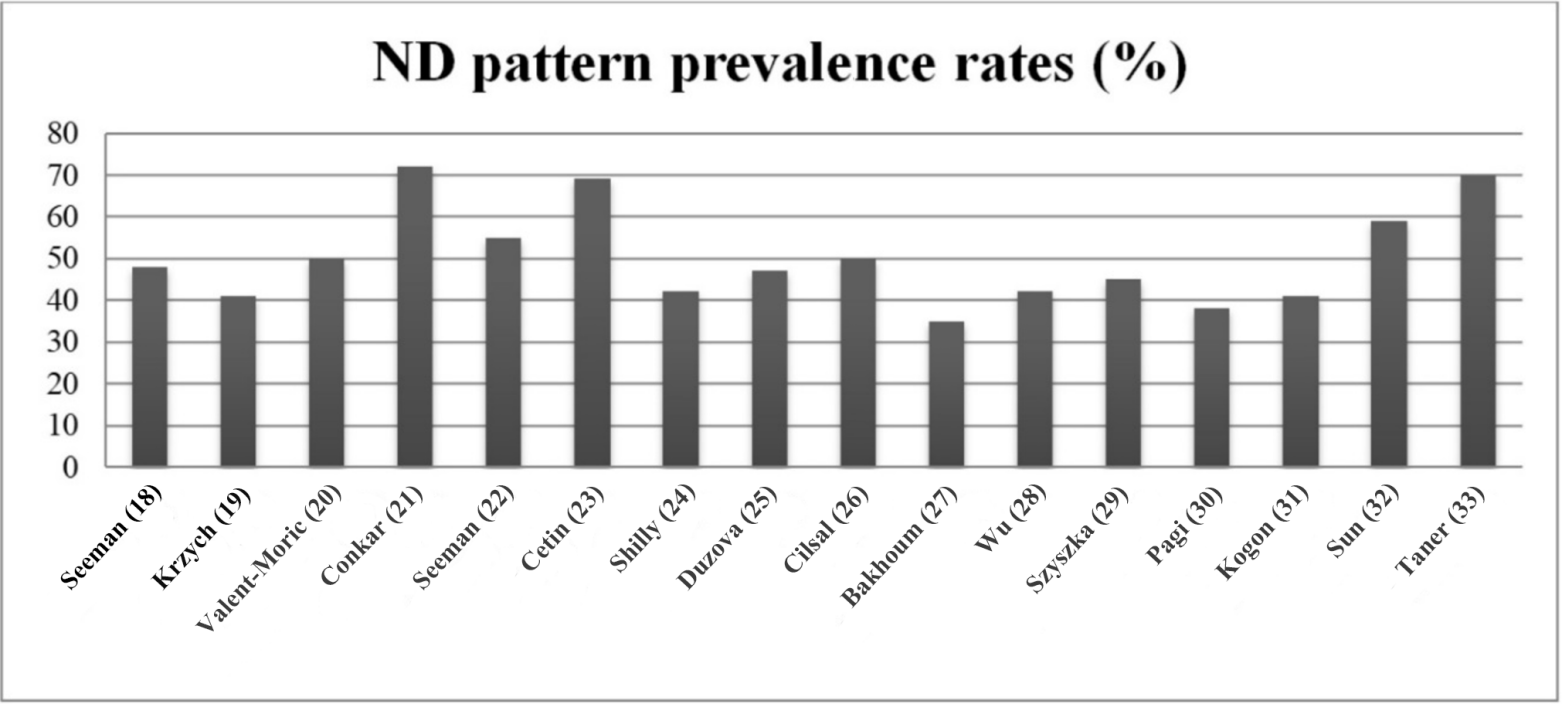
**Prevalence rates of the ND pattern in the selected studies**.

Table 2 (Ref. [18, 19, 20, 21, 22, 23, 24, 25, 26, 27, 28, 29, 30, 31, 32, 33]) summarizes some key aspects of the methods used in various studies to classify the ND phenotype. ABPM was performed on the non-dominant arm using validated devices in all the studies. The most widely used ABPM device was Spacelabs in its various versions (i.e., 90201, 90207, and 90217). Both patients’ diary-based criteria and wide or narrow fixed periods were used to define the daytime and nighttime bed rest periods. The length of the nighttime period varied across studies, whereas the exclusion of a transition period between day and night was reported in only one study [28]. The classification of the ND pattern, whose threshold was a drop <10% of the average nighttime BP compared to daytime BP, was substantially homogeneous across the studies and in line with the criteria used in hypertensive adults. Both systolic BP (SBP) and diastolic BP (DBP) were considered in the majority of studies. Fig. 2 illustrates the prevalence of ND patterns in hypertensive children and adolescents examined in the selected studies. The prevalence of reduced nocturnal BP fall in hypertensive children/adolescents varied widely, ranging from 35 to 72% (mean, 59%). Notably, the prevalence of this abnormal pattern was equal to or greater than 50% in approximately half of the reports. Four of the 16 studies included normotensive control individuals; however, only two of them provided data on the prevalence of the ND pattern in both groups, which was found to be lower in normotensive than in hypertensive subjects (29.5 and 29.8% versus 47.0 and 49.0%, respectively) [24, 31]. Two further studies revealed that patients with primary hypertension exhibited a larger nocturnal BP dip than those with secondary hypertension, which translated into a greater prevalence of the ND pattern in the latter (65.0 and 66.7% vs. 11.0 and 49.4%, respectively) [18, 20].

**Table 2.** **Summary of ambulatory blood pressure monitoring methodologic aspects in 16 studies addressing circadian variations in the setting of pediatric hypertension**.

Author (reference), year publication	Type of device	Day-night periods	Definition of non-dipping
Seeman *et al* [18], 2005	Spacelabs 90207 or 90217	daytime (8 AM to 8 PM)	SBP dip <10%
		nighttime (12 AM to 6 AM)	and/or DBP dip <10%
Krzych and Szydlowski [19], 2009	NIBP2 (Del Mar Reynolds Medical Ltd)	daytime (6 AM to 10 PM)	SBP dip <10%
		nighttime (10 PM to 6 AM)	
Valent-Moric *et al* [20], 2012	Mobilgraf M01100120 (I.E.M. GmbH, Stolberg)	n. a.	n. a.
Conkar *et al* [21], 2015	Spacelabs 90217	According to participants’ diary	SBP dip <10%
			or DBP dip <10%
Seeman *et al* [22], 2016	Spacelabs 90207 or 90217	According to participants’ diary	SBP dip <10%
			and/or DBP dip <10%
Cetin and Kavaz Tufan [23], 2019	Scanlight II/III	n. a.	SBP dip <10%
			and/or DBP dip <10%
Shilly *et al* [24], 2019	Spacelabs Model 90201‐IQ	n. a.	SBP dip <10%
			and DBP dip <10%
Düzova *et al* [25], 2019	Spacelabs 90207 or 90217	n. a.	n. a.
Cilsal [26], 2020	IEM-Mobil-O-Graph	n. a.	SBP dip <10%
			and DBP dip <10%
Bakhoum *et al* [27], 2021	Spacelabs 90217 or 90227	n. a.	SBP dip <10%
			and DBP dip <10%
Wu *et al* [28], 2022	DMS-ABP, DM Software Inc	daytime (9 AM to 9 PM)	SBP dip <10%
		nighttime (1 AM to 6 AM)	
Szyszka *et al* [29], 2022	Welch Allyn VSM Patient Monitor 300	n. a.	SBP dip <10%
			or DBP dip <10%
Pagi *et al* [30], 2023	Spacelabs 90217	n. a.	SBP dip <10%
			or DBP dip <10%
Kogon *et al* [31], 2024	OnTrak 90227 Spacelabs	According to participants’ diary	SBP dip <10%
			and/or DBP dip <10%
Sun *et al* [32], 2025	ABPM 7100, Welch Allyn	daytime (7 AM to 10 PM)	SBP dip <10%
		nighttime (10 PM to 7AM)	or DBP dip <10%
Taner *et al* [33], 2025	Meditech-04 (SunTech Medical Instruments)	n. a.	SBP dip <10%
			or DBP dip <10%

SBP, systolic blood pressure; DBP, diastolic blood pressure; n.a., not available.

Finally, no difference emerged when comparing the prevalence of ND pattern in studies conducted in children/adolescents with primary hypertension (7 studies, range 40–70%, mean 46.3%) with those carried out in their counterparts with CKD (5 studies, range 35–70%, mean 43.6%).

### 3.3 ND and HMOD

**Fig. 3.**
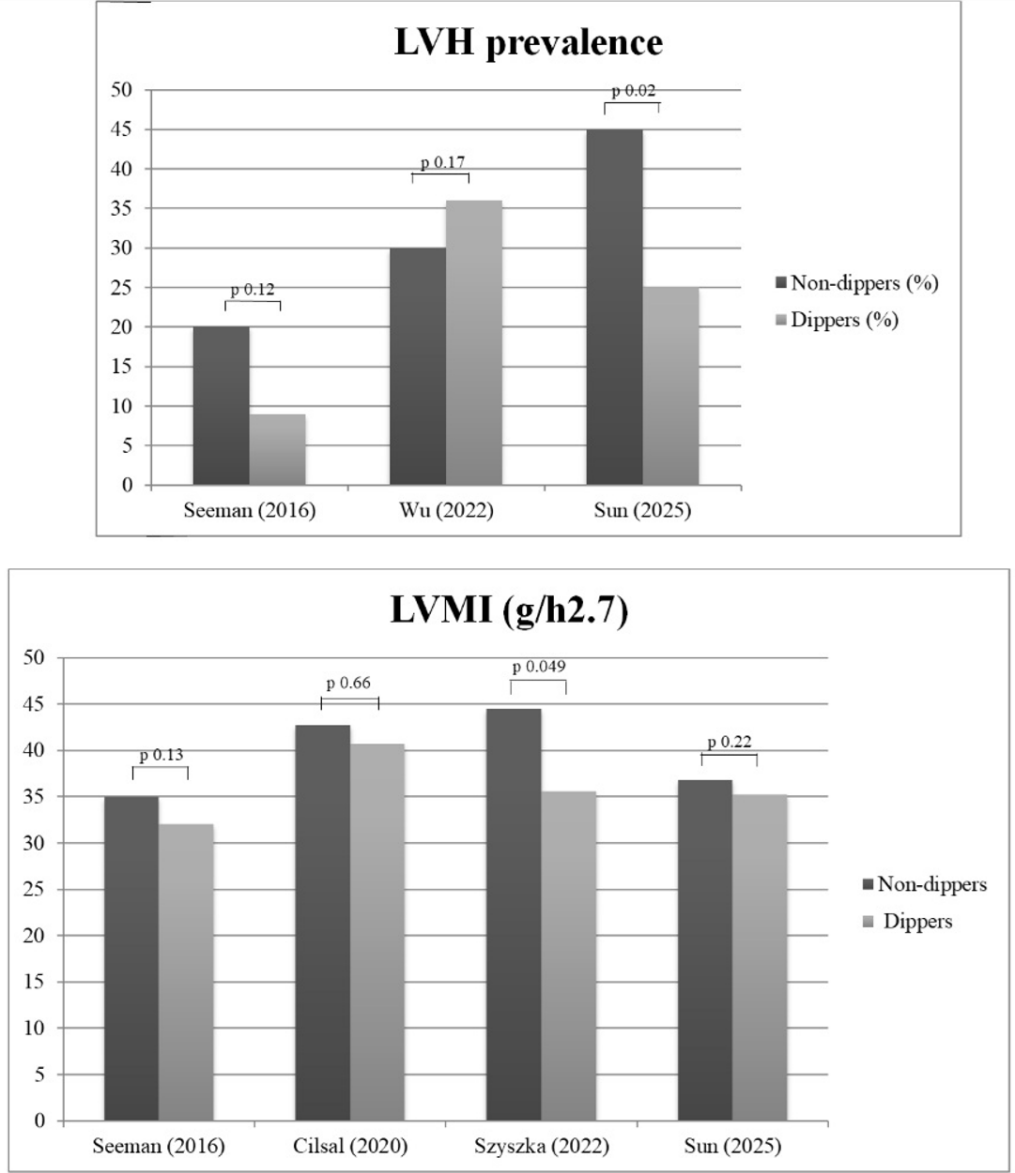
**Left ventricular structural changes in non-dippers versus dippers**. Prevalence of echocardiographic left ventricular hypertrophy in non-dippers (ND) versus dippers (D) in three studies providing categorical data on this cardiac phenotype (upper panel). Left ventricular mass index (LVMI) in four studies providing data on this continuous parameter (bottom panel).

Eight out of 16 studies provided HMOD data separately for dippers and non-dippers. Subclinical cardiac damage, defined by echocardiographic metrics as continuous (i.e., left ventricular mass index [LVMI]) or categorical variable (left ventricular hypertrophy [LVH]), was the most investigated marker. As summarized in Table 3 (Ref. [21, 22, 26, 27, 28, 29, 32, 33]), these studies reached different conclusions regarding early cardiac involvement in non-dippers; however, only three reports have shown a higher prevalence of LVH or increased LVMI associated with this condition [29, 32, 33], while the remaining studies found no difference (Fig. 3). Regarding markers of vascular damage (retinopathy, increased pulse wave velocity, and carotid intima-media-thickness [CIMT], data from three studies), no significant differences were detected. Proteinuria, but not microalbuminuria, was found to be higher in non-dippers [27, 29].

**Table 3.** **Hypertensive mediated organ damage (HMOD) in 8 studies providing data in childhood/adolescents with hypertension according to dipping (D), non-dipping (ND) status**.

Author (reference), year publication	Dippers number (prevalence male)	Non dippers number (prevalence male)	HMOD
Conkar *et al* [21], 2015	59 (n. a.)	23 (n. a.)	HMOD (Retinopathy, CIMT, LVMI) was detected in 66.1% of ND in 60.8% of D patients without reaching a statistically significant difference.
Seeman *et al* [22], 2016	63 (n. a.)	51 (n. a.)	LVMI adjusted for age and sex and LVH were not different in D and ND.
Cilsal [26], 2020	15 (67%)	15 (53%)	LVMI and PWV values were not different in D and ND.
Bakhoum *et al* [27], 2021	50 (68%)	27 (59%)	Significantly higher prevalence of proteinuria in ND.
Wu *et al* [28], 2022	189 (n. a.)	236 (n. a.)	LVH prevalence was not different in D and ND groups.
Szyszka *et al* [29], 2022	50 (64%)	62 (75%)	LVMI but not PWV, CIMT and MA was significantly higher in ND.
Sun *et al* [32], 2025	74 (77%)	51 (82%)	LVMI was not different, but LVH prevalence higher in ND (45%) than in D (25%).
Taner *et al* [33], 2025	99 (n. a.)	41 (n. a.)	LVH more frequent in ND (*p* = 0.04).

CIMT, carotid intima-media thickness; LVMI, left ventricular mass index; PWV, pulse wave velocity; ND, non-dipping; MA, microalbuminuria; LVH, left ventricular hypertrophy; n.a., not available.

### 3.4 Factors Related to ND Pattern

Table 4 (Ref. [18, 19, 23, 26, 29, 30, 31, 32]) summarizes a series of demographic, clinical, racial, and biochemical variables reported by some studies (identified with reference numbers) as factors implicated in the reduced nocturnal drop in BP. Male sex, obesity, and more severe hypertension emerged as independent determinants of the ND pattern. A comparison between white and African-American participants revealed that the latter group was more exposed to the risk of an abnormal nocturnal BP dip. Platelet alterations (i.e., increased mean platelet volume, distribution width, and platelet count), metabolic (i.e., hypertriglyceridemia), and inflammatory states (i.e., high-sensitivity C-reactive protein) have been associated with an ND pattern. Finally, a study performed in a large normotensive and hypertensive pediatric cohort showed that excessive sleep duration, based on age-specific cutoffs of the American Academy of Sleep Medicine, was associated with decreased nocturnal systolic and diastolic dipping.

**Table 4.** **Factors associated to non-dipping pattern in hypertensive children/adolescents as reported by studies cited as reference number (in brackets)**.

Factors associated to non-dipping pattern in hypertensive children/adolescents	Ref.
Male sex and more severe degree of hypertension	[18, 19]
Increased mean platelet volume and platelet count	[23]
Reduced heart rate variability	[26]
Excess body weight	[29]
Black ethnicity	[30]
Excessive sleep duration	[31]
Increased platelet distribution width, triglycerides and high sensitivity C-reactive protein	[32]

## 4. Clinical Implications 

The present review of 16 studies published since 2005 provides comprehensive and updated information on the prevalence of ND patterns in a large pooled population of children and adolescents with hypertension, showing that this abnormal BP phenotype is highly frequent in this setting, ranging from 35% to 72%, and affecting an average of 59% of participants. Indeed, comparing this finding with those reported by meta-analyses and studies conducted in adults and elderly hypertensive, general population cohorts, and normotensive children/adolescents, the reduced nocturnal BP fall in pediatric hypertension that emerged from our analysis appears to be a highly prevalent phenomenon.

Indeed, a systematic meta-analysis including a total of 3591 untreated adult hypertensive subjects from 23 studies (mean age range 41–72 years, 55% men), without heart failure, coronary heart disease, valve defects, or secondary causes of hypertension, documented that ND occurred in 36% of the cases [34]. Among the participants (mean age 69 years, 50% men, 48% treated with BP-lowering drugs) in the third survey of the Pressioni Arteriose Monitorate e Loro Associazioni (PAMELA) study, a prospective population-based observational survey, the ND pattern was identified in 35% of individuals [35]. As for the normotensive pediatric setting, Mezick *et al*. [36] examined a biracial cohort of 246 adolescents (mean age 15.7 years), free from CV or kidney disease not taking sleep, cardiovascular, or psychiatric medications and found that the ND pattern (defined by systolic BP or diastolic BP sleep-wake ratio >90) occurred in 33% of black and 20% of white participants, respectively, that is, a percentage rate substantially lower than that mentioned in the pooled population of hypertensive children/adolescents.

The high overall prevalence of ND revealed in this study can be ascribed to the fact that approximately half of the selected studies included children and adolescents with secondary hypertension and/or CKD. Among the clinical signs suspicious or suggestive of hypertension from secondary causes, a “reverse dipping” or ND profile at 24 h ABPM, not justified by other factors, is regarded as a possible red flag of this condition. In fact, many of the mechanisms underlying the most frequent forms of secondary hypertension (usually associated with higher BP values than essential hypertension), such as overactivated renin-angiotensin-aldosterone and the sympathetic nervous system, volume expansion, and sodium retention, may contribute to altering the physiological nocturnal drop in BP [37, 38, 39, 40]. Unfortunately, head-to-head comparison data aimed at investigating the prevalence of ND among essential versus secondary hypertension in children and adolescents are scarce and, as highlighted in our literature review, limited to a couple of studies [18, 20]. Therefore, the conclusion regarding a significantly greater prevalence of the ND pattern in pediatric patients with secondary hypertension requires further confirmation. On the other hand, it is useful to emphasize that even in adults with secondary hypertension, evidence regarding a definite pathophysiological link between the two entities is mixed [41, 42].

A comprehensive interpretation of the prevalence and clinical significance of alterations in the circadian rhythm of BP in hypertensive pediatric settings cannot ignore unsolved problems in this field of research. Although there are some differences between the various guidelines in the criteria used to define hypertension (i.e., different fixed diagnostic cutoff points for adolescents starting at age 13 or 16 years), there is a general consensus regarding the use of nomograms based on age, sex, and height in individuals below the aforementioned age thresholds [17, 43, 44]. In contrast, with regard to the dipper/ND classification, the same criterion adopted in the adult population, regardless of whether it identifies an abnormally reduced nocturnal drop according to systolic BP or diastolic BP sleep-wake ratio >90, has been extended without any variation by age and sex to the pediatric population and is currently used for clinical and research purposes. However, this metric does not consider the potential differences in the mechanisms underlying the reduction of nocturnal BP in children, adolescents, and adults. Indeed, the physiological mechanisms regulating nocturnal BP are multifactorial and extremely complex [45]. During sleep, there is deep readjustment of the mechanisms implicated in BP and heart rate regulation, including those mediated by the autonomic nervous system, kidney, and endocrine system. In turn, these mechanisms are influenced by a variety of factors, including physical activity, ambient temperature, season, evening meal, sleep architecture, microstructure, and duration [46, 47, 48].

With age, some aspects of the sleep–wake rhythm undergo widely known changes, such as lighter nocturnal sleep with more arousals, less slow-wave sleep, and occurrence of daytime naps [49]. Rapid eye movement (REM) sleep takes more time in children and adolescents than in adults. Unlike non-REM, REM sleep is characterized by high BP variability resembling wakefulness, associated with an increase in sympathetic activity to the skeletal muscles, which may alter the magnitude of nocturnal BP drop in young people [50]. These observations collectively support the view that using the <10% threshold to define nocturnal BP decline as abnormal may be questionable in the pediatric setting, and it would be preferable to consider the mean nocturnal BP as a more reliable diagnostic target according to the criteria recommended by the guidelines [16].

Many studies covered in this review have focused on the relationship between ND patterns and HMOD. This is because subclinical cardiac and vascular alterations in children and adolescents with hypertension may develop early and are relatively frequent. Before commenting on the results of the studies targeting the association between ND and HMOD considered in this review, some further general data are useful to briefly define the topic. LVH is a pivotal marker of HMOD in both children and young people. A large meta-analysis of the prevalence of this abnormal cardiac phenotype evaluated 51 study cohorts, including 5622 individuals with primary hypertension, 73% male participants, and a mean age of 13.6 years [51]. The prevalence of echocardiographic LVH varied from 20% in patients identified through community screening to 30% in those referred to specialty clinics, with a predominant LV eccentric pattern in the latter group. More recently, a systematic review of 39 studies with 3609 children and adolescents revealed that children with ambulatory hypertension had more LVH (odds ratio, 4.69), elevated LVMI (pooled difference, 5.13 g/m^2.7^), PWV (pooled difference, 0.39 m/s), and CIMT (pooled difference, 0.04 mm) than their normotensive counterparts did. The associations between ambulatory hypertension and LVMI and CIMT persisted following meta-regression, adjusting for body mass index (BMI) as a potential confounder [52]. At present, information on extracardiac damage, such as microalbuminuria and hypertensive retinopathy, remains limited. Kollias *et al*. [53] analyzed the data provided from five studies totaling 355 patients (mean age 13.1 years) and found a moderate association between diastolic ambulatory blood pressure (ABP) and urine albumin excretion. A study comparing peripapillary vessel density using optical coherence tomography angiography in children with high BP and healthy subjects demonstrated the presence of subthreshold microvascular alterations in the deep retinal capillary plexus without signs of hypertensive retinopathy [54].

Our review adds a new piece of information by highlighting the following: Most studies that investigated the relationship between ND pattern and HMOD have provided data on subclinical cardiac involvement through echocardiographic metrics. The cumulative prevalence of LVH in dippers and non-dippers reported in selected studies ranged from 10 to 37%, demonstrating that adverse phenotypes may affect one-tenth to over one-third of children/adolescents with hypertension. The prevalence rates of LVH and/or mean LVMI values were found to be completely superimposable in participants categorized as dippers/non-dippers in four studies; in contrast, in the remaining three studies, the presence of subclinical cardiac organ damage was higher in patients with the ND pattern. It is evident that these mixed results lead to the question of whether the blunted BP drop in the setting of pediatric hypertension could actually be a factor aggravating the presence of HMOD. Data on macro- and microvascular damage appear to be less conclusive due to their paucity and inconsistency: PWV (three studies), CIMT (two studies), and retinopathy (one study) showed no differences between the two groups. A higher amount of proteinuria was reported in ND patients compared to their counterparts with preserved nocturnal BP fall in a study conducted in the setting of hypertension with CKD [27], on the contrary no difference in microalbuminuria was observed in another study in patients with primary hypertension categorized according to the dipping status [29]. It should be noted that categorization of nocturnal BP phenotypes (i.e., dipping versus ND) based on single ABPM (as done in all selected studies) has been shown to have limited reproducibility over time, this is inherent to the fact that even small changes from one ABPM session to the next may result in a change in classification. Thus, to overcome this limitation the use of continuous variables in assessing night-time BP change may better reflect the relationship between the magnitude of the nocturnal BP drop and outcomes. This approach was performed in two of the selected studies and showed an inverse dose-response relationship between blood pressure drop and markers of organ damage (i.e., proteinuria and LVM index) [27, 29].

Finally, factors associated with the ND pattern have been investigated by a very limited number of studies, which beyond sex, BMI, ethnicity, and CV autonomic function have extended their focus to low-grade inflammation and less obvious variables such as platelet volume and count. Consistent with findings reported in adulthood, overweight, obesity, and black race were found to be related to the risk of the ND pattern [55, 56, 57]. Of potential interest is the line of research dedicated to the relationship between sleep structure and duration and circadian BP variations, which has revealed an association between excessive sleep duration and ND patterns [31]. This topic needs to be developed by considering the differences in sleep characteristics and lifestyles between children and adults.

## 5. Conclusion

Current evidence targeting the prevalence of the ND pattern and its association with HMOD in pediatric hypertension is based on a limited number of studies, heterogeneous in terms of design (retrospective/prospective), sample size, clinical setting and ethnicity. The high prevalence of reduced nocturnal BP falls documented in the selected studies, predominantly conducted in specialist centers, may not reflect real-world data in the entire pediatric population with elevated BP. Furthermore, the causal relationship between ND and HMOD remains largely undefined due to the cross-sectional nature of the studies and their conflicting results.

Future prospective studies in large representative pediatric populations with high BP are still needed to determine whether the classification of patients according to dipping or ND patterns adds useful clinical information to ambulatory BP diagnostic thresholds, namely mean nighttime BP. In this regard, the current knowledge gaps regarding specific diagnostic criteria for pediatric settings instead of those used in adults and the reproducibility of the ND pattern over time need to be filled.
